# Impact resistance of oil-immersed lignum vitae

**DOI:** 10.1038/srep30090

**Published:** 2016-07-18

**Authors:** Wei Yin, Lei Shan, Hongyu Lu, Yelong Zheng, Zhiwu Han, Yu Tian

**Affiliations:** 1 State Key Laboratory of Tribology, Tsinghua University, China; 2 Key Laboratory of Bionic Engineering (Ministry of Education, China), Jilin University, China

## Abstract

Biological materials immersed in vegetable and mineral oil, such as rattan armor and wooden sleepers, have been extensively used since ancient times because of their excellent mechanical properties. This study quantitatively investigated the viscoelasticity and tribological performance of lignum vitae immersed in poly-alpha-olefin (PAO) and tung oils (*Aleuritesfordii* Hemsl.) to reveal the mechanism of impact resistance. The acceleration of samples immersed in tung oil was higher than that of dry and PAO-immersed samples in the first impact. The elastic modulus of the samples immersed in tung oil increased slightly. The impact damage on the samples immersed in tung oil was reduced because of the low friction coefficient (0.07) resulted in a low wear rate. The extent of impact damage on the samples immersed in tung oil was approximately 34% and 58% lower than that on the dry and PAO oil-immersed samples, respectively, under an angle of 20° and a height of 10 cm. The impact damage on the PAO-immersed samples was reduced because of low friction coefficient. However, impact damage increased because of large elastic modulus. The findings of this study can serve as a reference for the application of modified biological materials with high strength and wear resistance.

Biological materials are typically multifunctional. However, many of them have been optimized to possess only a primary mechanical function^1^,^2^,^3^. Natural biofiber composites, such as rattan cane and wood, have been extensively utilized since ancient times. Rattan armor, a rattan cane create through vegetable oil immersion, provides strong protection against the cold^4^. This material exhibits high strength and good toughness; thus, it cannot be destroyed by swords and arrows. Rattan armor was extensively utilized in wars in ancient China. Wood exhibits characteristics similar to those of rattan cane, such as having simple elements and a sophisticated structure^5^,^6^,^7^. A wooden sleeper is wood processed by immersion in asphalt oil to obtain excellent mechanical properties, high wear resistance, and good anti-corrosion capability^8^,^9^. The manufacture of wooden sleepers has a less serious effect on climate change than the manufacture of steel or concrete. At present, wooden sleepers are widely used for technical and economical reasons^10^,^11^.

Rattan cane and wood are processed through oil immersion to enhance their mechanical properties. Impact and friction are two important factors during the loading process. The mechanical properties of rattan cane and wood, such as tensile, compressive, and flexural properties as well as related influencing factors, have been studied^12^,^13^,^14^. Wood exhibits anisotropic mechanical properties because of its anisotropic fiber structure. Its modulus of elasticity is affected by density, moisture content, temperature, microstructure, filaments, knot size, and location^15^,^16^. Researchers have shown considerable interest in the single tracheid stretching technique since it was reported^17^. This method is used to test the mechanical properties of wood, and numerous studies have been conducted on this method^18^,^19^. Fresh or decayed wood and its mechanical characterizations have been investigated, and a large number of experimental and numerical studies on dry wood, whose applications in construction and industries are extended, can be found in the literature^20^,^21^. The differences between fresh or decayed wood and dry wood are significant mainly because of the influence of the water content on the mechanical properties^22^,^23^.

Studies on the impact resistance of wood focused on impact with a pendulum or rock. The resistance to rock impact, energy dissipation capacity, and dynamic response of wood stems to impact with a pendulum have been studied^24^. The experimental results of these studies provided the definition of different impact types related to the occurrence of nonlinear processes associated with partial rupture of wood fibers. The physics controlling wood loading force and displacement are mainly associated with inertial effects during the early stages of impact. A finite element (FE) model of a live stem subjected to local dynamic loading has been proposed^25^. The FE model accounts for the wood’s multifiber feature and the dissymmetry of the mechanical response under tensile and compressive regimes. The simulations showed good agreement with the experimental measurements and the computational cost was reduced. The kinetic energy of a falling boulder along a slope was calculated in a previous study in consideration of the kinetic energy dissipated during impact^26^,^27^. The output of the simulation models was compared with the real dynamics of falling boulders in field tests through a digital video.

The tribological performance of wood–wood, wood–steel, and wood–polymer frictional pairs has been studied^28^,^29^,^30^. The moisture content, grain characteristic, and surface roughness of wood as well as loading and sliding speeds are important factors that affect wood friction^31^. Anisotropic friction behavior in wood occurs because of the vascular fiber orientation of this material^32^. Friction forces are minimized or maximized when the grain directions of two wood specimens are parallel each other or perpendicular, respectively^33^. The friction coefficients of wood initially increase sharply and subsequently stabilize with increasing arithmetic mean deviation of the surface profile^34^. However, the fundamental mechanism of oil-immersed wood and rattan cane has not been investigated yet. To date, no systematic and quantitative study that evaluated the effect of oil-immersed biofiber composites on mechanical and tribological properties is available.

Genuine lignum vitae (*Mesuaferrea* L.) exhibits high compressive and yield strengths^35^. Lignum vitae was modified in the current study through immersion in tung oil (*Aleuritesfordii* Hemsl.). Dry and poly-alpha-olefin (PAO)-immersed samples were utilized for comparative analysis. The modified mechanical and tribological properties of tung oil-immersed lignum vitae were also studied. Understanding the fundamental mechanism of the response of tung oil-immersed lignum vitae to external mechanical stimuli is important for the development of advanced functional materials.

## Results

### Microscopic structure of lignum vitae

**Figure 1:**
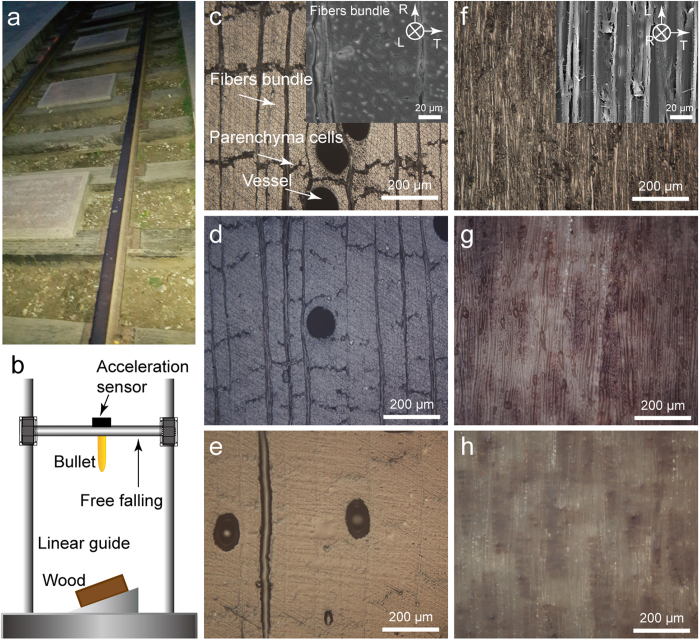
Applications of modified biological materials and the microscopic structure of lignum vitae in both cross and longitudinal sections. (**a**) Oiled wooden sleeper, photo taken in Linglong Park, Beijing, a relic park. (**b**) Schematic diagram of the mechanical impact test rig. (**c**) Cross section of dry lignum vitae. The inset was magnified via scanning electron microscopy (SEM). (**d,e**) Cross sections of PAO- and tung oil-immersed lignum vitae samples. (**f**) Longitudinal section of dry lignum vitae. The inset was magnified (**f**) via SEM. (**g,h**) Longitudinal sections of PAO- and tung oil-immersed lignum vitae samples, respectively.

The extensive application of modified biological materials, namely, wooden sleeper, is shown in Fig. 1a. To analyze the effect of oil on the impact resistance of the PAO- and tung oil-immersed lignum vitae samples, the impact of a pistol bullet on the surface of the lignum vitae samples was observed at different heights and angles. A schematic of the impact mechanical test rig is shown in Fig. 1b. The microscopic structures of dry, PAO-immersed, and tung oil-immersed lignum vitae samples in both cross and longitudinal sections are presented in Fig. 1c–h. The dry lignum vitae structure is layered with alternate distributions of fiber bundles and parenchyma cells (Fig. 1c,f). A rough fiber bundle is apparent at the micro level of lignum vitae (insets in Fig. 1c,f). Numerous vessels, fibers, and parenchyma cells exist in the lignum vitae samples. The vessels in each vascular bundle transport water and nutrients. The diameter of a lignum vitae fiber bundle is approximately 15 μm. The cell walls of lignum vitae are approximately 15 μm thick. The density and porosity of lignum vitae are approximately 1.12 g/cm^3^ and 30%, respectively. The moisture content of the dry lignum vitae sample is approximately 15%. The cross and longitudinal sections of the microscopic structure of PAO- and tung oil-immersed lignum vitae samples are shown in Fig. 1d–h. A layer of oil film (PAO or tung oil) is attached on the surface of cell walls. The oil fills in the fibers and parenchyma cells but not the vessels.

### Impact resistance mechanical property

**Figure 2:**
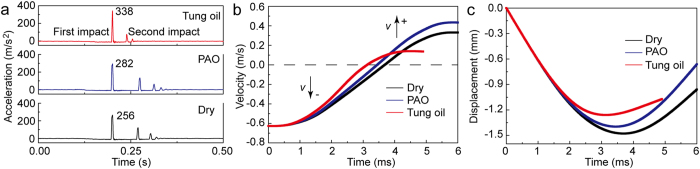
Representative impact mechanical property curves of dry, PAO-immersed, and tung oil-immersed lignum vitae samples. (**a**) Impact acceleration curves at a height and angle of 2 cm and 0°, respectively. (**b**) Velocity change curves during the first impact. (**c**) Impact displacement change curves during the first impact.

The representative impact acceleration curves of the dry, PAO-immersed, and tung oil-immersed lignum vitae samples are shown in Fig. 2a. The freely falling bullet repeatedly hit the surfaces of the samples and bounced off until it stopped. The velocity and impact displacement change curves during the first impact are shown in Fig. 2b,c, respectively. Downward movement was defined as a negative value. Impact velocity had a negative value in the beginning of impact and acquired a positive value when the bullet rebounded after impact to the maximum depth. The direction of impact acceleration was upward when the bullet impacted the surface of wood and rebounded. The first impact was less than 6 ms. Impact velocity initially decreased until it reached zero and subsequently increased for the three lignum vitae samples (dry, PAO-immersed, and tung oil-immersed) during the first impact. The increase in velocity caused the subsequent impact. Impact displacement initially increased and then decreased because of the elastic characteristic of lignum vitae during the first impact. The impact velocity and displacement of the tung oil-immersed sample are the smallest among the three samples.

**Figure 3:**
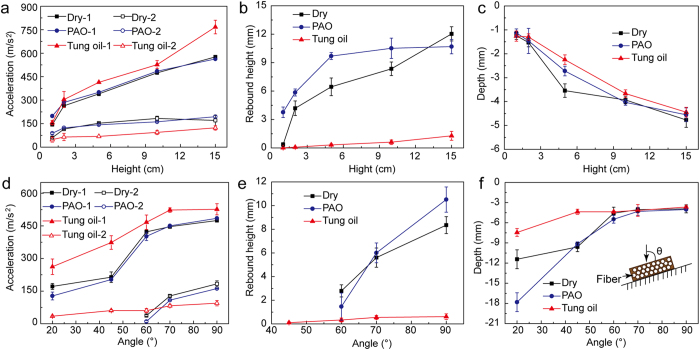
Impact mechanical properties. (**a**) First and second acceleration values at different impact heights. (**b**) Rebound height after the first impact at different heights. (**c**) Impact depth at different heights. (**d**) First and second acceleration values at different impact angles. (**e**) Rebound height after the first impact at different impact angles. (**f**) Impact depth at different impact angles. The inset in (**f**) is the impact direction of the impact tests at an impact height of 10 cm.

Figure 3 shows the mechanical properties of the three lignum vitae samples at different impact heights and angles. Acceleration, rebound height, and impact depth increased with impact height ranging from 2–15 cm. The acceleration of the tung oil-immersed lignum vitae samples is higher than that of dry and PAO-immersed lignum vitae samples in the first impact at different heights (Fig. 3a); however, the value is smaller than that in the second impact. The rebound height and depth of tung oil-immersed samples are smaller than those of the other samples after the first impact (shown in Fig. 3b,c, respectively). The acceleration of the tung oil-immersed lignum vitae samples is higher than that of the dry and PAO-immersed lignum vitae samples in the first impact at different angles (Fig. 3d). The rebound height and depth of the tung oil-immersed samplesare smaller than those of the other samples after the first impact (shown in Fig. 3e,f, respectively). Impact cracks at different impact angles are shown in Fig. S1. The impact depth of the tung oil-immersed samples decreased to a value less than 7.5 mm at an angle of 20° and height of 10 cm. This impact depth is approximately 34% and 58% smaller than that of the dry samples (about 11.4 mm) and samples immersed in PAO oil (about 17.8 mm), respectively. The internal pores of porous lignum vitae were filled with oil instead of air after immersed in oil. The deformation property of the oil within the pores was smaller than that of air; this condition resulted in increased impact acceleration after oil immersion. The lignum vitae samples immersed in tung oil can absorb much energy because of the large impact acceleration. This observation explains why oiled armor has small damage to people^36^.

### Mechanical properties and chemical composition

The mechanical properties of the samples were studied through dynamic mechanical analysis (DMA) and are shown in Table 1. The storage modulus of the lignum vitae samples increased after immersion in PAO and tung oil, whereas the loss modulus decreased. The loss factors (tan δ) of the PAO- and tung oil-immersed lignum vitae samples are smaller than that of the dry sample.

**Figure 4:**
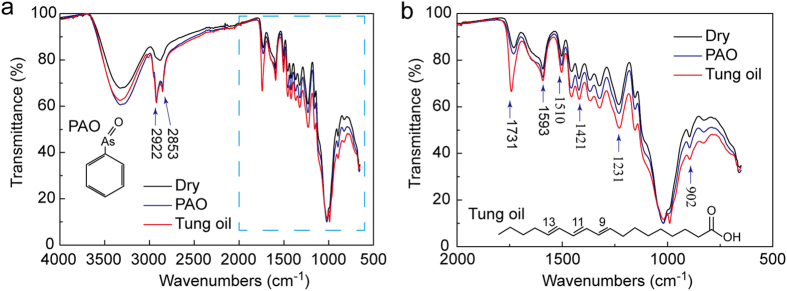
Infrared spectrograms of lignum vitae samples before and after oil immersion. (**a**) Infrared spectrograms of wave numbers 4000–500 cm^−1^. (**b**) Enlarged detail of (**a**) 2000–500 cm^−1^.

Figure 4 shows the infrared spectrograms of the lignum vitae samples at the range of 4000–500 cm^−1^. The absorption peaks at 3350 and 2922 cm^−1^ are attributed to the O−H and C−H bonds of the wood, respectively (Fig. 4a). The absorption peaks at the range of 2000–500 cm^−1^ are shown in Fig. 4b. The absorption peaks at 1510 and 1421 cm^−1^ are the characteristic peaks of the stretching vibration of benzene on behalf of lignin in the cell wall of the wood. The absorption peaks at 1731 and 1593 cm^−1^ are the characteristic peaks of the stretching vibration of xylan on behalf of hemicellulose in the cell wall of the wood. The absorption peaks at 902 and 1164 cm^−1^ are the characteristic peaks of cellulose in the cell wall of the wood. The absorption peaks at 2922 and 2853 cm^−1^ are the asymmetric and symmetric stretching vibration peaks of C–H, respectively. The two peaks are the widespread absorption peaks of hydrocarbons in PAO. The absorption peak at 1731 cm^−1^ is the stretching vibration peak of C = O in tung oil^37^.

### Tribological properties

**Figure 5:**
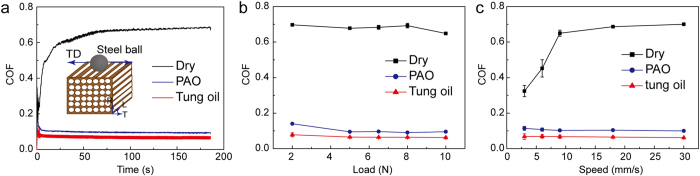
Tribological properties of the dry, PAO-immersed, and tung oil-immersed lignum vitae samples. (**a**) Representative friction coefficient curves of lignum vitae–steel ball. The friction coefficient during the first 80 s is the running-in period. The friction coefficient in the last 100 s is the result of the friction coefficient value. The inset shows the frictional directions of the lignum vitae–steel ball. The radial direction is perpendicular to the fiber cross-section direction. (**b**) Friction coefficient of lignum vitae under normal load values ranging from 2 N to 10 N. (**c**) Friction coefficient of lignum vitae under different friction speed values ranging from 3 mm/s to 30 mm/s.

Figure 5a shows the friction coefficient curves of dry, PAO- immersed, and tung oil-immersed lignum vitae samples under a load and speed of 5 N and 18 mm/s, respectively. The friction coefficients gradually increased and subsequently stabilized under dry friction. However, the friction coefficients of the PAO- and tung oil-immersed samples rapidly increased, gradually decreased, and eventually stabilized. Figure 5b shows the friction coefficients under different loads of dry, PAO-immersed, and tung oil-immersed lignum vitae samples. The friction coefficients under dry friction exhibited several changes as normal load is increased from 2–10 N. However, the friction coefficients of the oil-immersed samples are decreased slightly. The friction coefficients increased sharply as sliding velocity increased from 3–9 mm/s under dry friction, as shown in Fig. 5c. The friction coefficients remained constant when sliding velocity was higher than 9 mm/s. However, the friction coefficients of the PAO- and tung oil-immersed samples changed slightly as sliding velocity increased from 3–30 mm/s. The friction coefficient of the tung oil-immersed samples significantly decreased to a value less than 0.07 when the speed exceeded 9 mm/s under a load of 5 N. This friction coefficient is much smaller than those of the dry (about 0.70) and PAO-immersed (about 0.10) samples.

### Influence of oil on friction

**Figure 6:**
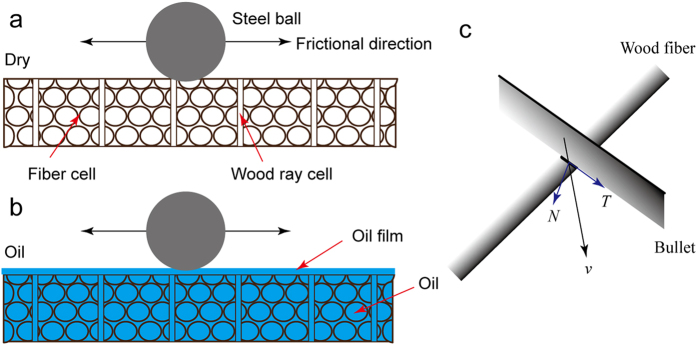
Schematics of the frictional properties. (**a**) Friction of the dry lignum vitae sample. (**b**) Influence of oil on the frictional properties of the oil-immersed lignum vitae samples. A lubrication film layer forms on the surface after the lignum vitae samples are immersed with PAO and tung oil. (**c**) Impact force (*N*) of the vertical direction and shear force (*T*) of the horizontal direction in an unrestricted impact process (*v*, movement direction of impact).

The friction coefficient peaks rapidly increased in the oil-immersed samples (Fig. 5a). The friction coefficient gradually decreased because of the oil film that formed on the friction interface, which indicates the presence of oil inside a hole in lignum vitae through deformation (shown in Fig. 6a,b). The friction coefficient stabilized over time. The conditions were virtually similar to those of boundary lubrication, and the observed low friction coefficient was nearly independent of load and speed. Oil was present as 3D supply from the holes of the lignum vitae; hence, the surface layer was readily replenished, and friction did not increase considerably with repeated traversals on the same track.

According to Amontons’ and Bowden’s theories^38^,^39^, contact peaks are compressed when plastic deformation occurs in lignum vitae. The contact area between the lignum vitae and the steel ball increases as normal load decreases^40^. The friction coefficient increases with normal load. However, internal friction or elastic hysteresis losses comprise a major portion of the resistance to slipping when a steel ball slips on lignum vitae. A frictional interface only occurs when deformation and elastic hysteresis loss are low. Therefore, the friction coefficient has a relatively small variation at different loads^41^,^42^,^43^. The frictional coefficient of the dry lignum vitae sample sharply increased as sliding velocity increased and then remained nearly constant afterward. By contrast, the frictional coefficients of the oil-immersed lignum vitae samples decreased gradually as sliding velocity increased. The oil present in the boundary lubrication film can absorb and release friction heat partially as the steel ball slides on the lignum vitae. The grinding cracks of the dry and PAO-immersed lignum vitae samples are shown in Fig. S2.

The surface force of the oil-immersed lignum vitae samples can be divided into impact force in the vertical direction and shear force in the horizontal direction (Fig. 6c). Shearing force can increase interface friction force and oil-immersed wood wear. However, interface friction force was reduced because of the low friction coefficient, and wear was reduced because of the oil film.

### Relationship among mechanical, frictional, and impact resistance properties

Studying the relationships among mechanical, tribological, and impact resistance properties is important to understand the impact resistance of the PAO- and tung oil-immersed lignum vitae samples. The relationship among mechanical, frictional, and impact resistance properties can be discussion with the following three cases.

(a) The friction force developed during rubbing is a superposition of an interfacial component (*F*_int_) and a deformation component (*F*_def_)^38^,^44^. The total friction force is presented as follows:


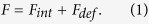


For the friction of biomaterial polymers and thin films, the deformation component may be ignored, and only the interfacial contribution is considered^38^. The ratio of the friction coefficients is approximated as follows:


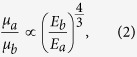


where *μ*_a_ and *μ*_b_ are the coefficients of the dry and oil-immersed lignum vitae samples, respectively, and *E*_a_ and *E*_b_ are the moduli of elasticity of the dry and oil-immersed lignum vitae samples, respectively.

Equation (2) shows that the ratio of the friction coefficients is inversely proportional to the ratio of the moduli of elasticity. A large moduli of elasticity means a small the friction coefficient. The modulus of elasticity of the lignum vitae increased after immersion in PAO and increased slightly after immersion in tung oil (Table 1). The friction coefficient of the lignum vitae increased after immersion in PAO because of the large modulus of elasticity. The small friction coefficient caused less damage in the PAO-immersed samples (Fig. 6c). The friction coefficient of tung oil immersed samples increased slightly as the modulus of elasticity increased slightly.

(b) The impact accelerations of the PAO-immersed samples were lower than those of the tung oil-immersed samples (Fig. 3a,d). The rebound height and impact depth of the PAO-immersed samples were large at different impact heights and angles and resulted in large damage. The degree of impact damage on two hard subjects is larger than that on soft and hard ones. The elasticity of the PAO-immersed samples increased significantly. The elasticity of the tung oil-immersed samples increased slightly, but their viscosity was significantly low. The degree of impact damage on the tung oil-immersed lignum vitae samples was lower than that on the dry and PAO oil-immersed lignum vitae samples. The property of impact resistance was reduced because of the increased elastic modulus.

(c) Impact depth was generated when the bullet hit the surface because of the high friction coefficient of the dry lignum vitae sample. However, the friction coefficients of the tung oil- and PAO-immersed lignum vitae samples were lower than that of the dry lignum vitae sample used as a stable lubrication film on the frictional interface. The bullet slid at the beginning of impact, and impact depth was generated after sliding. The surface of the tung oil- and PAO-immersed lignum vitae samples increased as a result of the process involved in deformation as the bullet passed over the lignum vitae. Impact damage was low when the bullet slid under a low friction coefficient (Figs 3f and 5b,c). Hence, the degree of impact damage on the tung oil-immersed samples was the lowest because of the lowest friction coefficient. The lower the friction coefficient is the smaller the impact depth is.

In summary, the impact damage on teh PAO-immersed samples was reduced because of the low friction coefficient. However, impact damages increases under a large elastic modulus. The elastic modulus of the tung oil-immersed samples increased slightly. The impact damage on the tung oil immersed samples was reduced because of the low friction coefficient.

The acceleration of the tung oil-immersed lignum vitae samples was higher than that of the dry and PAO-immersed lignum vitae samples in the first impact at different heights and angles. The elastic modulus of the tung oil-immersed samples increased slightly. A stable lubrication film layer was formed on the surface after oil immersion of the lignum vitae samples. The impact damage on the tung oil-immersed samples was reduced because of the low friction coefficient (0.07) and thus resulted in a low wear rate. The impact damage on the PAO-immersed samples was reduced because of the low friction coefficient. However, impact damage increased because of large elastic modulus. The results of this study can serve as a reference for the application of biological materials and their modified versions with high strength and wear resistance.

## Methods

### Microscopic structure analysis

Lignum vitae was prepared to determine the microstructural, mechanical, and tribological properties of the test samples. Mineral oil (poly-alpha-olefins, PAO) and vegetable oil (tung oil) were utilized to immerse lignum vitae under vacuum conditions. The viscosities of PAO and tung oils were approximately 19 and 35 *mpa.s* at 40 °C, respectively. The cells inside lignum vitae were filled with oil. Lignum vitae was soaked in oil for 48 h. The sample surfaces were carefully polished with sandpaper of different (roughness) grades. Four sandpaper grades, namely, 600^♯^, 1000^♯^, 4000^♯^, and 7000^♯^, and a polishing cloth (Struers Inc.) were used for the microstructural, mechanical, and tribological property tests to ensure that the lignum vitae surface had the same characteristics. Water was used as the polishing medium.

### Impact mechanical property test

An 88 pistol bullet was shot to the lignum vitae surface at a speed of 1.5 m/s. The surface roughness of the bullet was approximately 1.6 μm. The impact angles were 20°, 45°, 60°, 70°, and 90° at a height of 10 cm. The impact heights were 1, 2, 5, 10, and 15 cm at an angle of 90°. The dimensions of the lignum vitae samples were 30 mm × 15 mm × 10 mm. The bullet moved downward under the action of gravity along the guide rails. The lignum vitae samples could freely move in the vertical direction. Gravity was 1 kg. An accelerometer (CJMCU-ADXL001, China) was utilized to test the impact acceleration of the change process. The impact trace was cut from the middle, and the impact depth was measured from the side. Impact depth is the maximum depth of the penetration of the bullet. The initial impact velocity was


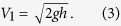


The velocity during impact was calculated as follows:


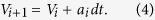


The displacement was obtained as follows:


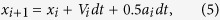


where *g* is the gravitational acceleration (9.8 m/s^2^), and *h* is the impact height (*i* = 1, 2, 3, ···, n).

### Chemical composition and mechanical property tests

The dry and PAO- and tung oil-immersed lignum vitae samples were studied through Fourier transform infrared (FT-IR, Magna-IR 750). The test parameters were subjected to a spectral scan of 200 times, with 4,000–500 cm^−1^ infrared range, 4 cm^−1^ resolution. KBr tableting test method was applied, and attenuated total reflection was determined.

A dynamic mechanical analyzer (TA Q800, USA) was used to test the dynamic mechanical properties of the dry and oil-immersed lignum vitae samples. Three specimens were prepared for each dynamic mechanical property test. Three-point bending properties were achieved through the experiments. The loading direction was perpendicular to the fiber direction. The dimensions of the DMA samples were 65 mm × 8 mm × 3 mm, and loading force had a frequency of 1 Hz. The test results were obtained from the average of three samples.

### Tribological tests

The dimensions of the friction test samples were 30 mm × 15 mm × 10 mm. Three specimens were prepared for each friction test. A micro-tribotester (UMT–2) was utilized as the friction test equipment. The reciprocating motion was based on the crank slider mechanism. A wood specimen was fixed onto a precise linear stage, which was connected to a rod driven by a motor. Upper balls (diameter: 4 mm) were reciprocated on the wood specimen at a stroke length of 3 mm under dry conditions. The reciprocating speed was fixed at 18 mm/s (180 rpm), and the normal load was increased from 2 N to 10 N to assess the loading effect. The amplitude was 3 mm. Loading was fixed at 5 N, whereas sliding speed varied from 3 mm/s (30 rpm) to 30 mm/s (300 rpm) to assess the effect of speed. The apparatus operated for 180 s at each speed and load value. Lignum vitae was immersed in PAO in a vacuum condition. The internal cells of lignum vitae were filled with oil. Lignum vitae was immersed in oil for approximately 100 h. The change trends of the tribological properties of the directions of the three lignum vitae samples against the steel ball are similar (Supplementary Figs S3, S4 and S5). Only the tribological properties of the tangential direction perpendicular to the fiber direction were studied. The grain and frictional directions were perpendicular.

## Additional Information

**How to cite this article**: Yin, W. *et al*. Impact resistance of oil-immersed lignum vitae. *Sci. Rep.* **6**, 30090; doi: 10.1038/srep30090 (2016).

## Supplementary Material

Supplementary Information

## Figures and Tables

**Table 1:** DMA results on mechanical properties.

	Storage Modulus(Es, × 10^4^MPa)	Loss Modulus(El, × 10^3^MPa)	Tan δ
Dry	8.26 (0.21)	8.95 (0.30)	0.11 (0.01)
PAO	11.91 (0.43)	3.47 (0.09)	0.03 (0.00)
Tung oil	8.59 (0.38)	3.19 (0.08)	0.04 (0.00)

The data in parentheses are standard deviations.
