# Supplementary material for: Impact resistance of oil-immersed lignum vitae
Source: Sci Rep. 2016 Jul 18;6:30090. doi: 10.1038/srep30090 (PMC4948022; doi:10.1038/srep30090)
Supplement: Supplementary Information [file srep30090-s1.pdf]

Additional Information

Impact resistance of oil-immersed lignum vitae

Wei Yin[1], Lei Shan[1], Hongyu Lu[1], Yelong Zheng[1], Zhiwu Han[2] & Yu Tian[1]

[1]State Key Laboratory of Tribology, Tsinghua University, China. [2]Key Laboratory of Bionic Engineering (Ministry of Education, China), Jilin University, China.

Correspondence and requests for materials should be addressed to Y.T. (email: tianyu@mail.tsinghua.edu.cn)

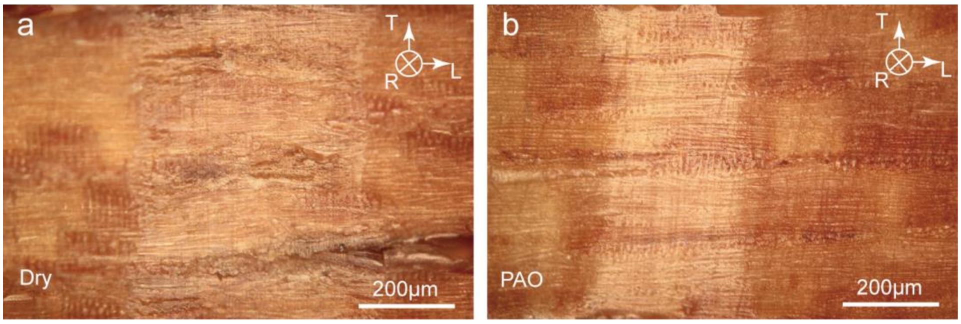

Figure S1 Grinding crack of the lignum vitae under a normal loads of 5 N and speeds of 18 mm/s.

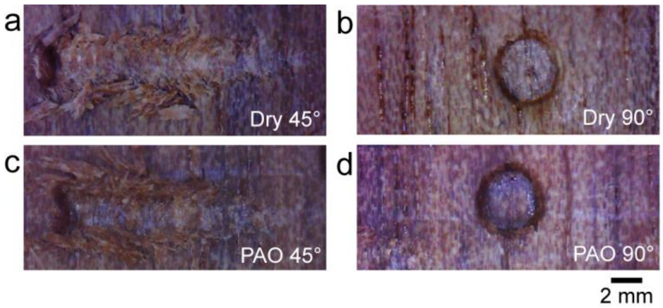

Figure S2 Impact crack through different impacting angles.

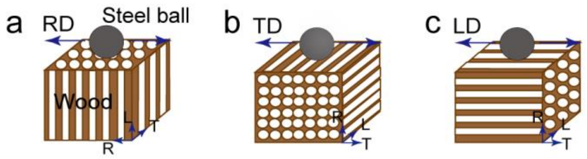

Figure S3 Lignum vitae–steel ball frictional directions. (a) The radial direction (RD) is perpendicular to the fiber cross section direction, (b) the tangential direction (TD) perpendicular to the fiber direction, and (c) the longitudinal direction (LD) along the fiber direction.

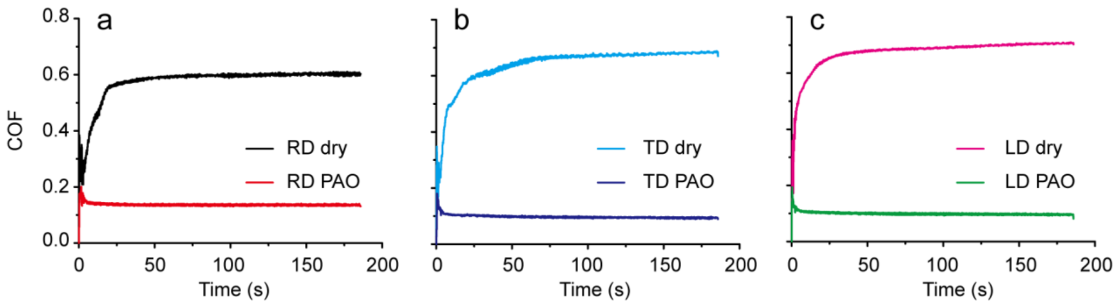

Figure S4 Friction coefficient curves and curves fitting of both dry and PAO immersed lignum vitae samples for different directions.

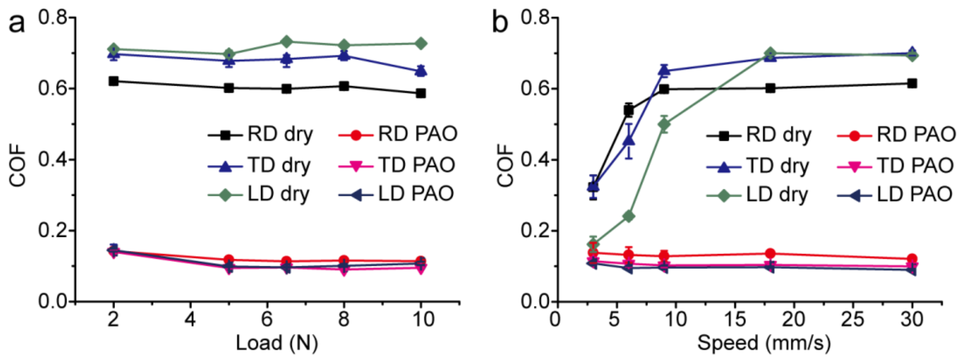

Figure S5 The coefficients of friction of lignum vitae under different loads and speeds. (a) Different loads from 2 to 10. (b) Different speeds from 3 to 30 mm/s. The friction coefficient of lignum vitae in the RD is lowest among the three directions for dry friction under different loads. The friction coefficient in the LD is the largest among the three directions. However, the friction coefficients in the three directions slightly differ for the PAO immersed samples. The normal load slightly affects the friction coefficient of lignum vitae.
